# Machine Learning-Driven Probability of Permanent Pacemaker Implantation After Transcatheter Aortic Valve Replacement

**DOI:** 10.3390/diagnostics16111720

**Published:** 2026-06-03

**Authors:** Marcel Abras, Daniela Bursacovschi, Ecaterina Pasat, Maria-Magdalena Vicol, Tatiana Abras, Lucia Mazur-Nicorici, Oleg Arnaut

**Affiliations:** 1 Department of Cardiology, Nicolae Testemitanu State University of Medicine and Pharmacy, MD-2004 Chisinau, Moldova; lucia.mazur@usmf.md; 2 Department of Interventional Cardiology, Public Healthcare Institution—Institute of Cardiology, MD-2025 Chisinau, Moldova; katea.pasat1991@gmail.com; 3 Department of Heart Failure, Public Healthcare Institution—Institute of Cardiology, MD-2025 Chisinau, Moldova; 4 Department of General Cardiology, Public Healthcare Institution—Institute of Cardiology, MD-2025 Chisinau, Moldova; pojogamagda@gmail.com; 5 Department of Endocrinology, Public Medico-Sanitary Institution Republican Clinical Hospital “Timofei Mosneaga”, MD-2025 Chisinau, Moldova; dr.tatiana.abras@gmail.com; 6 Department of Human Physiology and Biophysics, Nicolae Testemitanu State University of Medicine and Pharmacy, MD-2004 Chisinau, Moldova; oleg.arnaut@usmf.md; 7 Bioinformatics and Computational Medicine Laboratory, Nicolae Testemitanu State University of Medicine and Pharmacy, MD-2004 Chisinau, Moldova; 8 National Cancer Registry, Public Healthcare Institution—Oncological Institute, MD-2025 Chisinau, Moldova

**Keywords:** TAVR, permanent pacemaker implantation, prediction model

## Abstract

**Background/Objectives:** Permanent pacemaker implantation (PPI) remains one of the most common complications following transcatheter aortic valve replacement (TAVR). Identifying patients at increased risk for post-procedural conduction disturbances is clinically important for procedural planning and patient management. The aim of this study was to develop and evaluate a machine learning-based model for predicting the risk of PPI after TAVR. **Methods:** This prospective study was conducted between 2019 and 2025, and included 179 patients with severe aortic stenosis who underwent TAVR. Patient eligibility was determined by a multidisciplinary Heart Team based on clinical, echocardiographic, and imaging criteria. The primary endpoint was PPI occurring during hospitalization or within 30 days after the procedure. Statistical analyses were performed using RStudio (v. 2024.09.1+394)and Python (v.3.12.3), including comparative tests for continuous and categorical variables, receiver operating characteristic analysis to assess model performance, and SHapley Additive exPlanations (SHAP) to evaluate feature importance and model interpretability. **Results:** A total of 179 patients undergoing TAVR were included in the analysis. PPI occurred in 17 patients (9.5%) within 30 days after the procedure. A machine learning model was developed to predict post-TAVR PPI. The model demonstrated good predictive performance, with an overall accuracy of 0.944 and a weighted F1-score of 0.947. The confusion matrix showed that the model correctly classified 155 patients without PPI and 14 patients with PPI, with only a small number of false predictions. Explainability analyses using SHAP and permutation feature importance revealed that anatomical and procedural variables had the greatest impact on model predictions. The most influential predictors included valve size, right coronary sinus diameter, prosthetic valve diameter, and mean aortic annulus diameter. In contrast, baseline clinical variables such as left ventricular ejection fraction, previous myocardial infarction, and mean transaortic gradient showed a comparatively lower contribution to the prediction of PPI after TAVR. **Conclusions:** This study demonstrates that machine learning models can effectively predict the risk of PPI after TAVR. Anatomical characteristics of the aortic root and prosthesis-related parameters were the main determinants of PPI, whereas baseline clinical variables had a lower impact. The use of explainable artificial intelligence methods, such as SHAP analysis, may improve risk stratification and support procedural planning in patients undergoing TAVR.

## 1. Introduction

Transcatheter aortic valve replacement (TAVR) has rapidly evolved into the preferred treatment strategy for patients with severe aortic stenosis and, in many clinical scenarios, has largely replaced surgical aortic valve replacement [1,2]. However, alongside its widespread adoption, the procedure has introduced a distinct spectrum of complications that were less commonly encountered with conventional surgical approaches [3,4]. The incidence of permanent PPI after TAVR remains highly variable across published studies. A large meta-analysis including more than 29,000 patients reported PPI rates ranging from 6.7% to 39.2%, with a pooled incidence of approximately 19% (95% CI 16–21%) [5]. Data derived from contemporary large-scale registries further confirm the clinical impact of this complication. According to the Transcatheter Valve Therapy Registry, the overall rate of new PPI at 30 days following TAVR was approximately 11.3% [6]. Additional national registries and observational studies have reported median PPI rates between 9% and 14%, with considerable variation influenced by prosthesis design and procedural technique [7,8]. Registry-based analyses also highlight a broad incidence spectrum, ranging from approximately 2.3% to over 36%, reflecting differences in device generations, implantation strategies, and patient selection criteria [9]. Self-expanding prostheses have been consistently associated with higher rates of conduction disturbances compared to balloon-expandable valves, likely due to prolonged radial force exerted on the interventricular septum and the atrioventricular conduction system [10]. The need for PPI is not merely a procedural endpoint; it has been associated with longer hospital stay, increased healthcare costs, and potential long-term impact on left ventricular function and survival [11]. Although several clinical and anatomical predictors have been identified—such as pre-existing right bundle branch block, increased membranous septum length, deeper valve implantation, and extensive annular calcification—accurate risk stratification remains challenging [12,13]. Multimodality imaging, especially with pre-procedural computed tomography, has enhanced our ability to quantify anatomical risk. Notably, a shortened membranous septum—which places the conduction tissue closer to the plane of transcatheter valve deployment—is repeatedly associated with a higher likelihood of post-TAVR permanent pacemaker implantation. The length of the membranous septum and its relationship to the implantation depth of the prosthesis can help explain why certain patients develop high-grade atrioventricular block post-procedure, offering a structurally grounded predictor that integrates both patient anatomy and procedural variables [14]. In addition to CT measures, echocardiography (both transthoracic and transesophageal) contributes meaningfully to procedural planning and risk assessment. Echocardiographic delineation of left ventricular outflow tract geometry, valve annulus dimensions, and septal interactions with device positioning serves not only to guide sizing and trajectory but also correlates with conduction disturbance risk when these anatomical features predispose to deeper or asymmetric valve expansion. Echocardiographic data also provides real-time insights into prosthesis behavior and septal interplay during and immediately after deployment, complementing static CT measures [15,16]. Procedural factors continue to shape risk profiles as well. Implantation depth—especially when relatively deep within the left ventricular outflow tract—has been shown across several recent registries to increase the mechanical insult to conduction tissue, irrespective of valve design. While newer techniques such as cusp overlap projection and high implant strategies aim to minimize this influence, depth remains a salient, modifiable procedural parameter [1]. Furthermore, the characteristics of the prosthetic valve itself (for example, self-expanding versus balloon-expandable platforms, and valve sizing relative to native anatomy) influence device–tissue interactions that can predispose to conduction disruption [17,18,19]. Beyond anatomical and procedural considerations, recent registry data suggest that conduction disturbances and subsequent PPI are not merely procedural curiosities but have implications for clinical outcomes. Emerging analyses indicate associations between new PPI or new onset conduction abnormalities and increased heart failure hospitalization and mortality, especially as TAVR is increasingly performed in younger and lower-risk populations with longer life expectancy [20,21,22]. Taken together, the current landscape of evidence underscores the importance of an integrated risk assessment approach that combines baseline ECG characteristics, advanced imaging metrics (CT and echo), and careful procedural planning to stratify patients according to their likelihood of requiring a permanent pacemaker after TAVR. Such a multidimensional strategy holds promise not only for patient counseling and individualized procedural optimization but also for guiding post-procedural monitoring and longer-term rhythm management in this growing patient population.

## 2. Materials and Methods

### 2.1. Study Overview

The study was prospectively conducted at the Institute of Cardiology, Chisinau, Moldova. The study population consisted of 179 patients. Enrollment took place between 2019 and 2025, following approval from the Ethics Committee of the State University of Medicine and Pharmacy “Nicolae Testemitanu” (approval number 3/4.3, 19 March 2024). The study adhered to the principles of the Declaration of Helsinki. Written informed consent was obtained from all participants prior to inclusion.

Patients were screened for eligibility based on clinical, echocardiographic, and procedural criteria. All candidates were evaluated by a multidisciplinary Heart Team to determine suitability for transcatheter aortic valve implantation. Patients were eligible for inclusion if they met all of the following criteria: age ≥ 70 years; severe aortic stenosis defined as peak aortic jet velocity (Vmax) ≥ 4.0 m/s, or mean transvalvular gradient (GPmean) ≥ 40 mmHg, or aortic valve area (AVA) ≤ 1.0 cm^2^ (indexed AVA ≤ 0.6 cm^2^/m^2^); severe low-flow, low-gradient aortic stenosis (mean gradient < 40 mmHg) with supportive diagnostic confirmation; intermediate or high surgical risk, defined as EuroSCORE II > 2% and STS score > 4%; restricted mobility, severe frailty, or comorbid conditions potentially affecting postoperative rehabilitation; favorable vascular access for transfemoral TAVR. Patients were excluded if any of the following were present: mild or moderate aortic stenosis (Vmax ≤ 4.0 m/s or mean gradient ≤ 40 mmHg); left ventricular ejection fraction (LVEF) ≤ 30%; unfavorable valvular anatomy for TAVR, including short annulus-to-coronary ostium distance or severe symmetric calcification of the valve leaflets; patients with a prior PPI or other cardiac implantable electronic devices were excluded; active infective endocarditis; non-cooperative patients; refusal or inability to provide informed consent.

All patients underwent comprehensive clinical evaluation, laboratory testing, transthoracic echocardiography, and contrast-enhanced computed tomography for anatomical assessment of the aortic root, annulus, and vascular access. Surgical risk was calculated using the EuroSCORE II and the Society of Thoracic Surgeons risk model. TAVR procedures were performed according to current international guidelines using standard techniques. The type and size of prosthetic valve were selected based on multimodal imaging assessment and Heart Team decision-making. Implantation depth and procedural characteristics were documented for further analysis.

Patients who required PPI within the first 30 days following the TAVR procedure were included in the final outcome analysis. PPI was considered the primary endpoint when occurring during the index hospitalization or within 30 days after the procedure. Indications for PPI were established according to the current European Society of Cardiology guidelines on cardiac pacing and cardiac resynchronization therapy. These included: persistent high-grade atrioventricular block (second-degree Mobitz type II or third-degree atrioventricular block), advanced atrioventricular block with associated symptoms, alternating bundle branch block, and new-onset persistent high-grade conduction disturbances following TAVR. Additional indications comprised significant conduction abnormalities associated with hemodynamic instability, as well as documented pauses or symptomatic bradycardia not expected to resolve. In patients with new-onset left bundle branch block after TAVR, PPI was considered when accompanied by PR interval prolongation, QRS widening, intermittent high-grade atrioventricular block, or evidence of infranodal conduction delay confirmed by electrophysiological assessment, in accordance with contemporary ESC recommendations [23]. All PPI decisions were made by the institutional Heart Team in collaboration with electrophysiology specialists, following guideline-directed criteria and continuous rhythm monitoring.

### 2.2. Statistical Analysis

Statistical analyses were performed using RStudio (v. 2024.09.1+394) and Python (v. 3.12.3), ensuring full reproducibility of the analytical workflow. Continuous variables were summarized using measures of central tendency and dispersion, including mean, standard deviation, median, interquartile range, and observed range. Group comparisons for continuous data were conducted using the Mann–Whitney U test. Categorical variables were expressed as frequencies and percentages, accompanied by 95% confidence intervals. Relationships between categorical variables were assessed using Pearson’s Chi-squared test with Monte Carlo simulation (100,000 iterations). Statistical significance was defined as *p* < 0.05.

Machine learning methodology. Predictive modeling was performed as a supervised binary classification task. The dataset was structured into predictor variables (X) and a binary outcome (y). To ensure consistent and leakage-free analysis, model development was conducted using unified pipelines comprising three sequential stages: data preprocessing, class imbalance correction with the Synthetic Minority Over-sampling Technique (SMOTE), and classifier fitting. SMOTE was applied only within the training partitions of cross-validation in order to avoid information leakage and prevent optimistic bias in model performance estimation. Several candidate machine learning algorithms representing different modeling paradigms were compared, including class-weighted Logistic Regression, radial basis function Support Vector Machine, Random Forest, Extra Trees, Balanced Random Forest, and Multilayer Perceptron. Hyperparameters of candidate models were optimized within the cross-validation framework using systematic search procedures in order to identify the optimal model configuration while minimizing the risk of overfitting. Model performance was evaluated using stratified 3-fold cross-validation with shuffling and a fixed random seed (random_state = 42), ensuring preservation of the original class distribution across folds and reproducibility of results. Due to the relatively small number of outcome events, 3-fold cross-validation was preferred over higher-fold strategies in order to preserve an adequate distribution of PPI cases within each validation subset and to minimize instability related to sparse-event partitions. Given the exploratory design and limited event count, this approach was considered suitable for internal validation of the predictive model.

The evaluation metrics included balanced accuracy, F1-score, receiver operating characteristic area under the curve (ROC-AUC), and precision–recall area under the curve (PR-AUC). Because of the imbalanced class structure of the dataset, PR-AUC was used as the primary metric for model ranking and final model selection, as it provides a more informative measure of classifier performance under class imbalance conditions. Following model comparison, the best-performing algorithm was refitted on the full dataset using the same preprocessing and resampling pipeline to obtain the final predictive model. Final model performance was summarized using balanced accuracy, F1-score, ROC-AUC, PR-AUC, confusion matrix, and a detailed classification report including precision, recall, and F1-score for each class. According to the model comparison results, class-weighted Logistic Regression achieved the highest PR-AUC and was therefore selected as the final predictive model. To enhance model interpretability and facilitate clinical interpretation of predictor effects, model coefficients from the logistic regression model were transformed into Odds Ratios (OR) by exponentiation of regression coefficients. Odds ratios quantify the multiplicative change in the odds of the outcome associated with a one-unit increase in each predictor variable, allowing identification of both risk-enhancing and protective factors. In addition to classical statistical interpretation, model explainability was further investigated using SHAP (SHapley Additive exPlanations), a game-theoretic approach that quantifies the contribution of each feature to individual model predictions. SHAP analysis was used to estimate both global feature importance across the dataset and local feature contributions for individual predictions. Features were ranked according to their mean absolute SHAP values, allowing identification of the most influential predictors driving the model’s predictions. Feature importance analysis was also performed to evaluate the relative predictive contribution of variables in the model. Combining classical statistical interpretation (Odds Ratios) with explainable artificial intelligence techniques (SHAP) enabled a comprehensive understanding of both the magnitude and direction of predictor influence on the outcome, improving transparency and interpretability of the developed predictive model. All analyses were conducted using the Python programming language with open-source machine learning libraries including scikit-learn, imbalanced-learn, NumPy, pandas, and SHAP. Fixed random seeds and unified pipelines were used throughout the analysis to ensure full reproducibility of the modeling process.

## 3. Results

### 3.1. General Characteristics of the Study Population

We conducted a prospective analytical cohort study between 2019 and 2025, including 179 patients who underwent transcatheter aortic valve replacement. In the overall study population, the median age was 75.0 years (IQR 6.0). Women accounted for 60.9% (109 patients; 95% CI 54–68%) of the cohort, while men represented 39.1% (70 patients; 95% CI 32–46%). A history of previous myocardial infarction was reported in 16.8% (95% CI 11–22%) of patients. ECG analysis revealed the following findings in patients from the overall study population: atrial fibrillation was present in 23.5% (95% CI 17–30%) of the study population. Sinus rhythm was documented in 82.7% (95% CI 77–88%) of patients, while atrial fibrillation and other cardiac rhythms were less frequent. The median baseline heart rate was 70.0 bpm (IQR 18.0). Signs of left ventricular hypertrophy were detected in 57.5% (95% CI 50–65%) of participants. Left bundle branch block was present in 16.2% (95% CI 11–22%), and right bundle branch block in 6.7% (95% CI 3.0–10%) of patients. Analysis of medications administered prior to the TAVR procedure that could potentially affect cardiac rhythm and heart rate revealed that: 79.9% (95% CI 74–86%) of patients were receiving beta-blockers, and 4.5% (95% CI 1.4–7.5%) were treated with cardiac glycosides. Majority of patients had a severe aortic valve calcification attested in 63.1% (95% CI 56–70%), moderate calcification in 27.4% (95% CI 21–34%), and mild calcification in 9.5% (95% CI 5.2–14%) of cases. Most patients had a tricuspid aortic valve (94.4%; 95% CI 91–98%), while bicuspid morphology was observed in 5.6% (95% CI 2.2–9.0%). With regard to the implanted prosthetic valve, self-expanding prosthetic valves were used in 69.8% (95% CI 63–77%) of procedures, whereas balloon-expandable valves were implanted in 30.2% (95% CI 23–37%). Balloon predilatation was performed in 59.8% (95% CI 53–67%) of patients, and post-dilatation was required in 36.9% (95% CI 30–44%) of procedures. It should be noted that the median left ventricular ejection fraction was 58.0% (IQR 7.0), indicating overall preserved systolic function.

After presenting the general characteristics of the study population, we further analyzed and compared the data according to PPI status. The first group consisted of patients without PPI (No PPI) (*n* = 162), while the second group included patients who required PPI implantation (*n* = 17). Continuous variables were expressed as mean ± standard deviation (SD) and compared using the independent samples t-test. A *p*-value < 0.05 was considered statistically significant. Overall, no statistically significant differences were observed between patients with and without PPI in terms of baseline demographics, cardiovascular comorbidities, preprocedural medication with potential effects on heart rhythm or heart rate, or the majority of baseline ECG and echocardiographic parameters (all *p* > 0.05). Notably, the main between-group differences were related to procedural characteristics: self-expanding valves were used more frequently in patients who required PPI (94.1% vs. 67.3%; *p* = 0.024). In addition, patients who underwent PPI had a lower cardiac index (2.6 [IQR 0.6] vs. 2.8 [IQR 0.8]; *p* = 0.047), data presented in Table 1.

### 3.2. Development of a Machine Learning Model for Predicting Permanent Pacemaker Implantation

Based on these findings, the transition to a machine learning-based analytical approach was considered justified. Given that no significant differences were identified between groups in terms of baseline clinical, ECG, echocardiographic characteristics and majority aspects of technical characteristics of the procedure using conventional statistical methods, it is likely that the relationship between preprocedural variables and the need for PPI after TAVR is multifactorial and driven by complex, non-linear interactions that may not be adequately captured by traditional univariate or regression-based analyses.

To identify potential predictors of post-procedural PPI following TAVR, variables derived from the cardiovascular clinical profile, medical history, pharmacological therapy, electrocardiographic findings, anatomical imaging, and procedural characteristics were selected. In addition, technical parameters related to the implanted prosthetic valve were incorporated into the predictive model. Clinical cardiovascular variables included previous myocardial infarction, use of beta-blockers and cardiac glycosides (digitalis), and the presence of atrioventricular block or intraventricular conduction disturbances (LBBB, RBBB). Echocardiographic parameters comprised left ventricular ejection fraction, aortic valve area, and mean transaortic gradient. Anatomical and imaging-derived measurements included ascending aorta diameter, aortic annulus diameter, perimeter and area, mean annular diameter, coronary artery heights (right and left), diameters of the left, right, and non-coronary sinuses, cusp heights (LCC, RCC, NCC), and calcium score. Coronary anatomy variables such as atherosclerotic lesions, left main coronary artery stenosis, circumflex artery stenosis, and coronary dominance pattern (right, left, or balanced) were also analyzed. Procedural and prosthesis-related parameters included aortic valve type, prosthetic valve diameter, and valve size. Candidate variables were selected based on clinical relevance and previously reported associations with post-TAVR conduction disturbances. Because several anatomical measurements were potentially correlated, variables were reviewed during preprocessing to avoid excessive redundancy among highly overlapping features. No formal dimensionality reduction method was applied, given the exploratory nature of the study.

**Figure 1:**
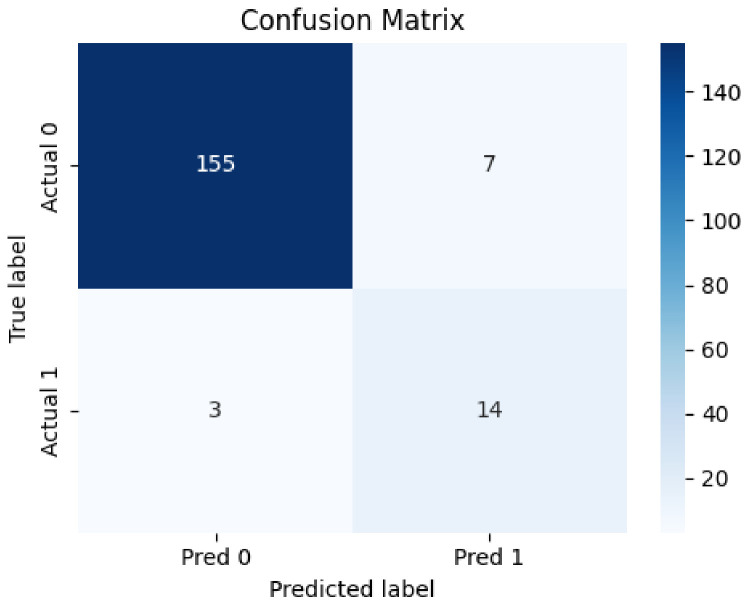
Confusion matrix of the predictive model for PPI.

An analysis of absolute and relative frequencies for the development of the predictive model for post-TAVR PPI revealed that this complication occurred in 9.5% of the studied patients. This proportion indicates a moderate class imbalance within the dataset; it allowed the application of standard classification methods for predictive modeling, with appropriate consideration of class distribution during model development. To evaluate the predictive performance of the developed machine learning model for PPI after TAVR, a confusion matrix analysis was performed (Figure 1).

As shown in the confusion matrix, the predictive model correctly identified 155 patients who did not require PPI following TAVR (true negatives) and 14 patients who subsequently underwent implantation (true positives). The model incorrectly predicted PPI in 7 patients who did not require it (false positives), while failing to identify 3 patients who ultimately needed a pacemaker after the procedure (false negatives). These findings indicate that the model demonstrates good discriminative ability in identifying patients at risk for post-procedural PPI, with a relatively low number of missed events despite the low incidence of the outcome in the study population (17 out of 179 patients). Correspondingly, the classification report showed a precision of 0.981 and recall of 0.957 for patients who did not require PPI (patients who did not require permanent pacemaker implantation), and a precision of 0.667 with a recall of 0.824 for those who required implantation (patients who required post-procedural pacemaker implantation). Given the class imbalance within the dataset, model performance was interpreted primarily using class-specific metrics rather than overall accuracy alone. The model achieved a recall of 0.824 for the PPI class, indicating that most patients requiring post-procedural pacemaker implantation were correctly identified, with only three false-negative cases. Precision for the PPI class was 0.667, reflecting the presence of false-positive predictions, which may have implications for clinical applicability. Overall accuracy and weighted F1-score remained high (0.944 and 0.947, respectively), although these metrics should be interpreted cautiously in the context of imbalanced outcome distribution.

**Figure 2:**
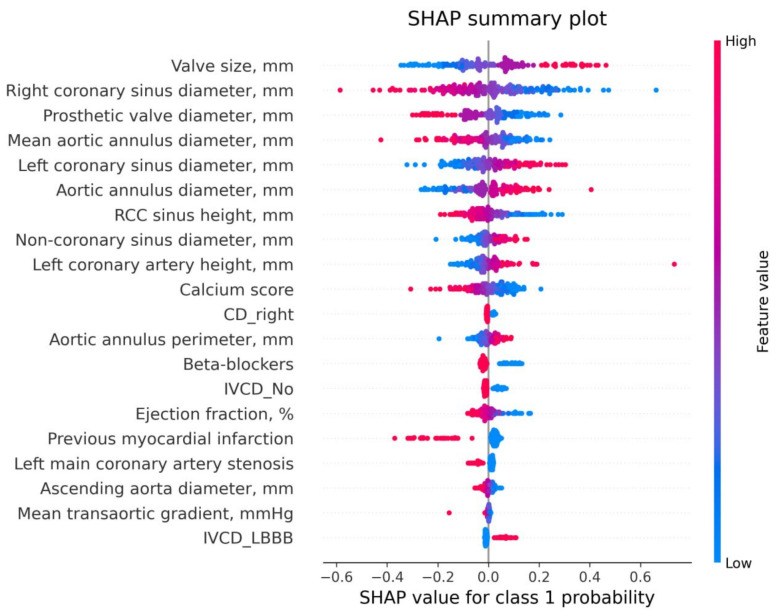
SHAP-based feature importance analysis for PPI prediction.

To further interpret the contribution of individual variables to the model’s predictions, SHapley Additive exPlanations (SHAP) analysis was performed (Figure 2). The SHAP summary plot illustrates the relative importance and direction of impact of each feature on the prediction of PPI following TAVR. In this plot, each dot represents an individual patient, with the position on the x-axis indicating the SHAP value, corresponding to the impact of a given feature on the model output. Positive SHAP values indicate an increased likelihood of post-procedural PPI, whereas negative values suggest a reduced predicted risk. Feature values are color-coded, with red representing higher values and blue indicating lower values. Among the analyzed variables, valve size, right coronary sinus diameter, prosthetic valve diameter, and mean aortic annulus diameter demonstrated the greatest impact on model predictions. Higher values of valve size and prosthetic valve diameter were generally associated with an increased predicted risk of PPI. Similarly, anatomical parameters such as aortic annulus diameter and coronary sinus dimensions contributed significantly to risk stratification. In contrast, clinical variables including ejection fraction, previous myocardial infarction, and mean transaortic gradient showed a comparatively lower influence on the model output. Overall, the SHAP analysis suggests that anatomical and procedural characteristics play a more prominent role than baseline clinical variables in predicting the need for PPI after TAVR.

**Figure 3:**
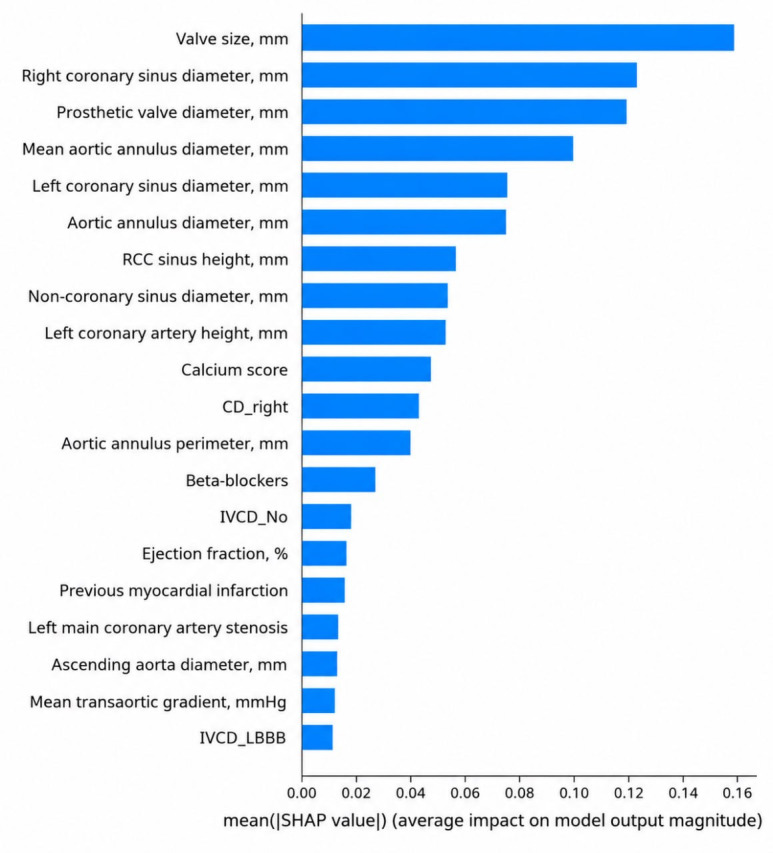
Feature importance based on mean absolute SHAP values for the prediction of permanent pacemaker implantation.

To quantify the overall importance of each variable in the predictive model, the mean absolute SHAP values were calculated and are presented in Figure 3.

This bar plot illustrates the average impact of each feature on the model output magnitude across the study population. Higher mean absolute SHAP values indicate a greater contribution of a given variable to the prediction of PPI following TAVR. Valve size demonstrated the highest overall importance in the predictive model, followed by right coronary sinus diameter, prosthetic valve diameter, and mean aortic annulus diameter. Additional anatomical variables, including left coronary sinus diameter, aortic annulus diameter, and coronary sinus height, also showed a substantial contribution to model predictions. In contrast, clinical variables such as ejection fraction, previous myocardial infarction, and mean transaortic gradient had a relatively limited impact on the predicted risk of post-procedural PPI. These findings further support the predominance of anatomical and procedural characteristics over baseline clinical parameters in determining the likelihood of PPI after TAVR.

**Figure 4:**
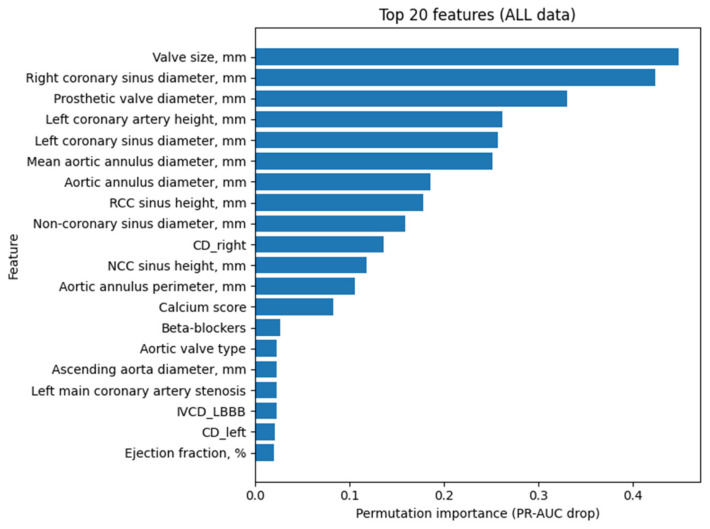
Top 20 features according to permutation importance.

To better understand which variables the model relied on most when predicting the need for PPI after TAVR, permutation feature importance analysis was performed (Figure 4). This approach evaluates the relevance of each predictor by measuring the extent to which model performance decreases when the values of that variable are randomly shuffled. In our analysis, valve size emerged as the most influential factor, followed by right coronary sinus diameter and prosthetic valve diameter. Several additional anatomical parameters, including left coronary artery height, left coronary sinus diameter, and mean aortic annulus diameter, also showed a meaningful contribution to the predictive performance of the model. In contrast, clinical characteristics such as ejection fraction, prior myocardial infarction, and beta-blocker therapy had only a minor impact. Overall, these findings suggest that anatomical and procedural features play a more prominent role than baseline clinical variables in determining the likelihood of PPI following TAVR.

## 4. Discussion

PPI remains one of the most frequent complications following transcatheter aortic valve implantation. The ability to accurately predict the risk of post-procedural PPI is therefore clinically relevant, as it may improve patient selection, procedural planning, and post-procedural monitoring strategies. Recent advances in artificial intelligence and machine learning have provided new opportunities for developing predictive models that integrate clinical, imaging, and procedural variables to estimate the risk of conduction disturbances after TAVR [24]. In the present study, we developed and evaluated a predictive machine learning model for PPI following TAVR and explored the relative contribution of anatomical, clinical, and procedural variables. The performance of the model, illustrated by the confusion matrix, demonstrated good discriminatory capability in identifying patients who required PPI. The relatively low number of false positives and false negatives indicates that the model may have practical utility in identifying patients at increased risk of conduction disturbances after the procedure. One of the strengths of the current analysis lies in the explainability of the model. Using SHAP analysis, we were able to identify and quantify the contribution of individual variables to the prediction of PPI. According to the SHAP summary plot, valve size represented the most influential variable, followed by right coronary sinus diameter, prosthetic valve diameter, and mean aortic annulus diameter. These findings are consistent with previous studies demonstrating that anatomical characteristics of the aortic root and prosthesis–annulus interaction play a key role in the development of conduction disturbances after TAVR [25,26]. Furthermore, the global feature importance analysis based on mean absolute SHAP values and permutation importance confirmed the dominant influence of anatomical parameters on the model output. Variables related to aortic root geometry, including coronary sinus diameters, annular dimensions, and coronary artery heights, were among the most relevant predictors. This observation is supported by previous research showing that anatomical proximity between the prosthesis and the conduction system, particularly in the region of the membranous septum and left ventricular outflow tract, may predispose patients to atrioventricular block requiring PPI [27,28]. Another important observation from our model is the contribution of clinical and procedural factors such as calcium score, beta-blocker therapy, and baseline conduction disturbances. Previous studies have also reported that conduction abnormalities and calcification burden are significant predictors of PPI after TAVR. In particular, pre-existing conduction disorders and extensive valvular calcification have been associated with an increased risk of injury to the conduction system during valve deployment [29,30]. Recent investigations have also explored the integration of multimodal data sources, including computed tomography, electrocardiography, echocardiography, and clinical parameters, to enhance predictive accuracy. For example, El Ouahidi et al. developed a machine learning model incorporating CT-derived anatomical measurements alongside clinical variables, demonstrating improved prediction of PPI risk after TAVR [25]. Similarly, artificial intelligence models based on ECG data have been shown to identify patients at increased risk of conduction disturbances with promising predictive performance [29,31]. Our findings align with these reports and further highlight the importance of anatomical features of the aortic root and prosthesis selection in predicting conduction disturbances after TAVR. The identification of valve size and sinus dimensions as major predictors suggests that procedural planning and device selection may play a key role in mitigating the risk of post-procedural PPI. SHAP analysis suggested that larger annular and prosthetic dimensions were generally associated with increased predicted PPI risk; however, the present dataset did not allow reliable determination of clinically applicable cutoff values. Future larger-scale studies may further clarify potential nonlinear relationships and interaction effects among anatomical and procedural predictors. By identifying the most influential predictors and providing interpretable outputs through SHAP analysis, such models may support clinicians in identifying patients at higher risk of conduction disturbances and optimizing procedural strategies.

## 5. Conclusions

In this study, we developed and evaluated a machine learning-based predictive model for PPI following transcatheter aortic valve implantation. The model demonstrated good predictive performance and provided clinically meaningful insights into the factors associated with post-procedural conduction disturbances.

Explainability analyses using SHAP and permutation importance highlighted that anatomical parameters of the aortic root and prosthesis-related measurements—particularly valve size, coronary sinus dimensions, and aortic annulus characteristics—were the most influential predictors of PPI. In addition, clinical factors such as calcium burden, baseline conduction abnormalities, and certain medical therapies also contributed to the predictive model, although to a lesser extent. Our findings emphasize the importance of detailed anatomical assessment and procedural planning in reducing the risk of conduction disturbances after TAVR. Moreover, the integration of explainable machine learning approaches may support personalized risk stratification and help clinicians identify patients at higher risk for post-procedural permanent pacemaker implantation. Future studies including external validation and the incorporation of additional imaging and electrophysiological parameters are needed to further improve predictive accuracy and facilitate the clinical implementation of such models in routine TAVR practice.

## 6. Limitations

Several limitations should be acknowledged. Although the overall cohort size was acceptable, the number of PPI events was relatively limited (*n* = 17), which may influence the stability of predictive estimates given the number of variables included in the machine learning analysis. To improve model robustness, cross-validation and SMOTE-based balancing techniques were applied; however, the findings should still be interpreted within the exploratory context of the study. In addition, SHAP-derived feature importance rankings may be influenced by sample-specific variability. Given the low number of positive outcomes and marked class imbalance, calibration results should be interpreted with caution. The out-of-fold Brier score was worse than the prevalence-only baseline, suggesting suboptimal calibration of the predicted probabilities. Therefore, the model should not be interpreted as providing reliable individualized absolute risk estimates without external validation in a larger dataset. Although the model may retain some ability to discriminate between higher- and lower-risk patients, the estimated absolute probabilities are likely unstable due to the limited number of events, the use of SMOTE and class weighting, and the potential for overfitting in a small-sample setting. Therefore, the proposed model should be considered a preliminary predictive framework that requires further validation in larger multicenter cohorts.

Although the model demonstrated encouraging performance within the present cohort, its generalizability to other populations and clinical settings remains uncertain. Therefore, the proposed model should be considered exploratory and hypothesis-generating, and further validation in larger independent multicenter cohorts is required before potential clinical implementation. Formal calibration analysis was not performed; therefore, agreement between predicted and observed outcomes could not be assessed. This should be addressed in future validation studies.

Also, future studies integrating additional imaging parameters, particularly detailed CT-based measurements of the membranous septum and conduction system proximity, may further improve predictive accuracy. Despite these limitations, the present study highlights the potential of explainable machine learning approaches for risk stratification in TAVR patients.

## Figures and Tables

**Table 1:** General characteristics of the patients.

	No PPI *n* = 162 ^1^	95% CI ^2^	PPI *n* = 17 ^1^	95% CI ^2^	Statistic Test ^3^	*p*-Value ^3^
**Age**	76.2 (4.7) 75.0 (6.0) 70.0 89.0	75, 77	77.6 (5.5) 76.0 (9.0) 70.0 88.0	75, 80	1183	0.3
**Sex**					0.03	>0.9
Feminine	99 (61.1%)	54%, 69%	10 (58.8%)	35%, 82%		
Masculine	63 (38.9%)	31%, 46%	7 (41.2%)	18%, 65%		
**Previous myocardial infarction**	29 (17.9%)	12%, 24%	1 (5.9%)	0.00%, 17%	1.6	0.3
**Atrial fibrillation**	37 (22.8%)	16%, 29%	5 (29.4%)	7.8%, 51%	0.37	0.6
**Body mass index**	29.1 (4.8) 29.3 (6.2) 19.5 41.8	28, 30	31.9 (5.6) 31.3 (5.5) 23.8 46.9	29, 35	999	0.063
**Renal dysfunction**	25 (15.4%)	9.9%, 21%	3 (17.6%)	0.00%, 36%	0.06	>0.9
**Beta-blockers**	129 (79.6%)	73%, 86%	14 (82.4%)	64%, 100%	0.07	>0.9
**Cardiac glycosides (digitalis)**	8 (4.9%)	1.6%, 8.3%	0 (0.0%)	0.00%, 0.00%	0.88	0.6
**Cardiac rhythm**					2.9	0.4
atrial fibrillation	23 (14.2%)	8.8%, 20%	5 (29.4%)	7.8%, 51%		
PPI	2 (1.2%)	0.00%, 2.9%	0 (0.0%)	0.00%, 0.00%		
sinus rhythm	136 (84.0%)	78%, 90%	12 (70.6%)	49%, 92%		
**Heart rate**	72.1 (12.6) 70.0 (18.0) 50.0 118.0	70, 74	67.5 (8.5) 65.0 (12.0) 59.0 85.0	63, 72	1682	0.13
**Signs of left ventricular hypertrophy**	95 (58.6%)	51%, 66%	8 (47.1%)	23%, 71%	0.84	0.4
**Atrioventricular block**					0.27	>0.9
No	148 (91.4%)	87%, 96%	16 (94.1%)	83%, 100%		
Yes, gr. I	12 (7.4%)	3.4%, 11%	1 (5.9%)	0.00%, 17%		
Yes, gr. II	1 (0.6%)	0.00%, 1.8%	0 (0.0%)	0.00%, 0.00%		
Yes, gr. III	1 (0.6%)	0.00%, 1.8%	0 (0.0%)	0.00%, 0.00%		
**Intraventricular conduction disturbances**					3.8	0.14
LBBB	26 (16.0%)	10%, 22%	3 (17.6%)	0.00%, 36%		
No	127 (78.4%)	72%, 85%	11 (64.7%)	42%, 87%		
RBBB	9 (5.6%)	2.0%, 9.1%	3 (17.6%)	0.00%, 36%		
**Ascending aorta diameter, mm**	37.0 (3.4) 37.0 (5.0) 28.0 49.0	36, 38	36.8 (3.5) 38.0 (3.0) 31.0 45.0	35, 39	1401	>0.9
**Aortic annulus diameter, mm**	22.3 (2.0) 22.0 (3.0) 17.0 28.0	22, 23	23.1 (1.2) 23.0 (2.0) 21.0 25.0	23, 24	1031	0.085
**Left atrium diameter, mm**	47.4 (5.8) 46.0 (7.0) 16.0 68.0	47, 48	46.8 (5.9) 46.0 (7.0) 37.0 58.0	44, 50	1463	0.7
**Left atrial volume, ml**	94.9 (34.6) 82.7 (40.3) 34.8 267.3	89, 100	90.9 (34.7) 82.7 (40.3) 43.1 165.8	73, 109	1462	0.7
**Left atrial volume index, ml/m^2^**	51.0 (18.9) 46.6 (18.6) 19.8 130.5	48, 54	47.2 (16.7) 44.1 (22.2) 24.6 81.4	39, 56	1540	0.4
**LV end-diastolic diameter**	50.9 (5.7) 50.0 (7.0) 39.0 71.0	50, 52	50.4 (4.3) 49.0 (5.0) 44.0 60.0	48, 53	1450	0.7
**LV end-diastolic volume**	132.3 (32.3) 132.0 (47.0) 70.0 227.0	127, 137	124.2 (25.5) 118.0 (37.0) 90.0 185.0	111, 137	1573	0.3
**LV end-systolic diameter**	34.8 (6.9) 34.0 (8.0) 21.0 64.0	34, 36	36.1 (7.6) 35.0 (10.0) 27.0 56.0	32, 40	1265	0.6
**LV end-systolic volume**	56.1 (24.9) 53.0 (30.8) 24.0 168.0	52, 60	54.1 (21.5) 51.0 (18.0) 27.0 115.0	43, 65	1408	0.9
**Interventricular septum thickness, mm**	14.6 (2.1) 14.0 (3.0) 11.0 22.0	14, 15	14.5 (1.6) 15.0 (3.0) 12.0 17.0	14, 15	1366	>0.9
**LV mass, g**	289.8 (63.1) 281.2 (91.5) 169.4 461.8	280, 300	276.5 (38.2) 275.8 (40.3) 206.4 365.4	257, 296	1495	0.6
**LV mass index, g/m^2^**	155.7 (35.0) 148.6 (49.1) 91.7 254.0	150, 161	144.9 (20.9) 143.7 (24.5) 111.5 189.9	134, 156	1572	0.3
**Relative wall thickness**	0.5 (0.1) 0.5 (0.1) 0.3 0.9	0.49, 0.51	0.5 (0.1) 0.5 (0.1) 0.4 0.6	0.45, 0.53	1394	>0.9
**Ejection fraction, %**	57.4 (7.8) 58.0 (7.0) 25.0 69.7	56, 59	57.0 (7.5) 60.0 (6.0) 37.0 70.0	53, 61	1431	0.8
**Right ventricle diameter, mm**	29.5 (4.5) 29.0 (6.0) 20.0 48.0	29, 30	29.2 (2.7) 30.0 (5.0) 25.0 34.0	28, 31	1322	0.8
**Right atrium diameter, mm**	45.6 (4.5) 45.0 (5.0) 32.0 60.0	45, 46	45.4 (4.2) 46.0 (4.0) 38.0 56.0	43, 48	1340	0.9
**E/e′ ratio**	11.9 (4.4) 10.6 (6.4) 4.9 32.0	11, 13	12.9 (4.7) 12.4 (7.4) 7.5 23.4	10, 15	1225	0.5
**Peak transaortic gradient, mmHg**	82.7 (20.5) 79.7 (25.8) 16.5 160.0	80, 86	88.4 (22.7) 86.5 (27.6) 58.1 139.6	77, 100	1198	0.4
**Mean transaortic gradient, mmHg**	56.0 (35.8) 51.8 (19.8) 11.1 473.0	50, 62	53.9 (13.7) 49.2 (12.6) 37.0 87.1	47, 61	1368	>0.9
**Peak velocity, m/s**	4.5 (0.6) 4.5 (0.7) 2.0 7.2	4.4, 4.6	4.7 (0.6) 4.6 (0.8) 3.8 5.9	4.4, 5.0	1205	0.4
**Stroke volume, mL**	75.1 (15.8) 75.0 (22.0) 42.0 148.0	73, 78	68.7 (7.9) 68.0 (8.0) 55.0 83.0	65, 73	1762	0.058
**Cardiac output**	5.4 (1.4) 5.3 (1.6) 2.2 11.1	5.2, 5.6	4.8 (0.9) 4.8 (1.2) 3.2 7.1	4.4, 5.3	1737	0.077
**Cardiac index**	2.8 (0.7) 2.8 (0.8) 1.4 6.1	2.7, 2.9	2.5 (0.5) 2.6 (0.6) 1.8 3.6	2.3, 2.8	1781	**0.047**
**Coronary dominance**					4.5	0.11
balanced	18 (11.1%)	6.3%, 16%	4 (23.5%)	3.4%, 44%		
left	13 (8.0%)	3.8%, 12%	3 (17.6%)	0.00%, 36%		
right	131 (80.9%)	75%, 87%	10 (58.8%)	35%, 82%		
**Atherosclerotic lesions**					2.5	0.5
normal coronary arteries	67 (41.4%)	34%, 49%	8 (47.1%)	23%, 71%		
single-vessel disease	13 (8.0%)	3.8%, 12%	3 (17.6%)	0.00%, 36%		
three-vessel disease	65 (40.1%)	33%, 48%	5 (29.4%)	7.8%, 51%		
two-vessel disease	17 (10.5%)	5.8%, 15%	1 (5.9%)	0.00%, 17%		
**History of bypass surgery**	6 (3.7%)	0.80%, 6.6%	0 (0.0%)	0.00%, 0.00%	0.65	0.6
**Left main coronary artery stenosis**	35 (21.6%)	15%, 28%	1 (5.9%)	0.00%, 17%	2.4	0.2
**Circumflex artery stenosis**	70 (43.2%)	36%, 51%	6 (35.3%)	13%, 58%	0.39	0.6
**Aortic valve type**					1.1	0.6
Bicuspid	10 (6.2%)	2.5%, 9.9%	0 (0.0%)	0.00%, 0.00%		
Tricuspid	152 (93.8%)	90%, 98%	17 (100.0%)	100%, 100%		
**Aortic annulus perimeter, mm**	77.3 (7.8) 76.5 (10.6) 25.4 94.0	76, 79	77.9 (5.8) 77.9 (6.8) 67.3 88.6	75, 81	1283	0.6
**Aortic annulus area, mm^2^**	461.2 (87.6) 450.0 (120.2) 23.6 683.9	448, 475	465.6 (71.6) 451.5 (99.5) 344.7 615.0	429, 502	1303	0.7
**Right coronary artery height, mm**	16.8 (2.7) 16.7 (3.2) 10.9 26.4	16, 17	17.0 (2.3) 16.8 (2.0) 13.6 22.7	16, 18	1341	0.9
**Left coronary artery height, mm**	13.6 (3.2) 13.0 (3.9) 7.1 26.2	13, 14	18.8 (20.3) 14.3 (4.9) 9.6 97.0	8.3, 29	1195	0.4
**Left coronary sinus diameter, mm**	32.0 (3.6) 31.7 (4.6) 23.4 43.0	31, 33	32.2 (2.0) 32.0 (2.4) 29.6 37.0	31, 33	1227	0.5
**Right coronary sinus diameter, mm**	30.9 (3.4) 30.8 (4.2) 22.7 42.2	30, 31	29.2 (3.9) 29.3 (4.3) 17.1 33.0	27, 31	1687	0.13
**Non-coronary sinus diameter, mm**	31.5 (3.5) 31.0 (4.5) 19.7 40.5	31, 32	30.8 (4.8) 30.8 (4.0) 15.8 37.2	28, 33	1387	>0.9
**LCC sinus height, mm**	20.9 (3.9) 21.0 (4.2) 9.2 35.0	20, 21	19.6 (4.6) 21.0 (4.0) 11.0 26.9	17, 22	1536	0.4
**RCC sinus height, mm**	21.1 (3.7) 21.7 (4.4) 10.8 32.2	21, 22	19.9 (3.9) 21.0 (4.4) 12.0 26.8	18, 22	1595	0.3
**NCC sinus height, mm**	21.4 (3.2) 21.0 (3.6) 15.0 36.0	21, 22	132.5 (460.1) 21.0 (2.7) 18.0 1918.0	-104, 369	1378	>0.9
**Sinotubular junction diameter**	28.7 (3.8) 28.2 (4.6) 21.3 47.0	28, 29	28.5 (2.9) 28.0 (2.6) 23.0 34.1	27, 30	1343	0.9
**Calcification grade**					1.7	0.5
High	104 (64.2%)	57%, 72%	9 (52.9%)	29%, 77%		
Moderate	44 (27.2%)	20%, 34%	5 (29.4%)	7.8%, 51%		
Reduced	14 (8.6%)	4.3%, 13%	3 (17.6%)	0.00%, 36%		
**Calcium score**	3168.6 (1918.8) 2700.0 (2464.3) 795.4 12,006.0	2871, 3466	2456.3 (1257.4) 2116.0 (1811.0) 518.0 4678.0	1810, 3103	1640	0.2
**Mean LVOT diameter, mm**	24.2 (2.5) 24.0 (3.4) 18.9 31.0	24, 25	23.8 (2.1) 23.9 (3.2) 20.9 28.0	23, 25	1505	0.5
Unknown	1		0			
**Minimum aortic annulus diameter, mm**	21.5 (2.5) 21.4 (2.9) 15.5 33.7	21, 22	21.6 (3.4) 21.3 (2.8) 17.8 31.6	20, 23	1522	0.5
**Maximum aortic annulus diameter, mm**	27.6 (2.6) 27.3 (3.6) 22.0 35.2	27, 28	27.4 (2.1) 27.1 (2.5) 24.0 30.8	26, 28	1382	>0.9
**Mean aortic annulus diameter, mm**	24.5 (2.3) 24.2 (3.3) 19.8 34.4	24, 25	24.5 (1.9) 24.5 (1.5) 21.0 28.2	24, 25	1336	0.8
**Porcelain aorta**	5 (3.1%)	0.42%, 5.7%	0 (0.0%)	0.00%, 0.00%	0.54	>0.9
**Prosthetic valve diameter, mm**	27.6 (3.4) 27.0 (4.0) 20.0 34.0	27, 28	27.0 (2.8) 26.0 (4.0) 24.0 35.0	26, 28	1527	0.5
**Valve type**					5.3	0.024
balloon-expandable	53 (32.7%)	25%, 40%	1 (5.9%)	0.00%, 17%		
self-expanding	109 (67.3%)	60%, 75%	16 (94.1%)	83%, 100%		
**Valve size, mm**	27.6 (3.4) 27.0 (4.0) 20.0 34.0	27, 28	28.3 (2.6) 29.0 (2.0) 25.0 34.0	27, 30	1155	0.3
**Balloon predilatation**	94 (58.0%)	50%, 66%	13 (76.5%)	56%, 97%	2.2	0.2
**Post-dilatation**	59 (36.4%)	29%, 44%	7 (41.2%)	18%, 65%	0.15	0.8
**Repositioning**					11	0.14
0	123 (75.9%)	69%, 83%	11 (64.7%)	42%, 87%		
1	22 (13.6%)	8.3%, 19%	3 (17.6%)	0.00%, 36%		
2	10 (6.2%)	2.5%, 9.9%	2 (11.8%)	0.00%, 27%		
3	6 (3.7%)	0.80%, 6.6%	0 (0.0%)	0.00%, 0.00%		
4	1 (0.6%)	0.00%, 1.8%	0 (0.0%)	0.00%, 0.00%		
5 and more	0 (0.0%)	0.00%, 0.00%	1 (5.9%)	0.00%, 17%		

Note: ^1^ Mean (SD), Median (IQR), Min Max; *n* (%), ^2^ CI = Confidence Interval. ^3^ Wilcoxon rank sum test; Pearson’s Chi-squared test with simulated *p*-value (based on 1 × 10^5^ replicates).

## Data Availability

The data presented in this study are available on request from the corresponding author due to patient confidentiality and data protection regulations.

## Abbreviations

The following abbreviations are used in this manuscript:

LVEF	Left ventricular ejection fraction
PPI	Permanent pacemaker implantation
SHAP	SHapley Additive exPlanations
TAVR	Transcatheter aortic valve replacement
